# Design and Assessment of Bird-Inspired 3D-Printed Models to Evaluate Grasp Mechanics

**DOI:** 10.3390/biomimetics9040195

**Published:** 2024-03-26

**Authors:** Pavan Senthil, Om Vishanagra, John Sparkman, Peter Smith, Albert Manero

**Affiliations:** Limbitless Solutions, University of Central Florida, 12703 Research Parkway, Suite 100, Orlando, FL 32826, USA; p.senthil@limbitless-solutions.org (P.S.); o.vishanagra@limbitless-solutions.org (O.V.);

**Keywords:** biomimicry, prostheses, 3D printing

## Abstract

Adapting grasp-specialized biomechanical structures into current research with 3D-printed prostheses may improve robotic dexterity in grasping a wider variety of objects. Claw variations across various bird species lend biomechanical advantages for grasping motions related to perching, climbing, and hunting. Designs inspired by bird claws provide improvements beyond a human-inspired structure for specific grasping applications to offer a solution for mitigating a cause of the high rejection rate for upper-limb prostheses. This research focuses on the design and manufacturing of two robotic test devices with different toe arrangements. The first, anisodactyl (three toes at the front, one at the back), is commonly found in birds of prey such as falcons and hawks. The second, zygodactyl (two toes at the front, two at the back), is commonly found in climbing birds such as woodpeckers and parrots. The evaluation methods for these models included a qualitative variable-object grasp assessment. The results highlighted design features that suggest an improved grasp: a small and central palm, curved distal digit components, and a symmetrical digit arrangement. A quantitative grip force test demonstrated that the single digit, the anisodactyl claw, and the zygodactyl claw designs support loads up to 64.3 N, 86.1 N, and 74.1 N, respectively. These loads exceed the minimum mechanical load capabilities for prosthetic devices. The developed designs offer insights into how biomimicry can be harnessed to optimize the grasping functionality of upper-limb prostheses.

## 1. Introduction

### 1.1. Motivation

A key area of development in the state of upper-limb prostheses is to improve prosthetic functionality [1]. A driving cause of high rejection or abandonment rates, estimated to be between 20 and 40 percent in recent decades, is prosthetic users finding their devices unreliable in interacting with objects in their day-to-day lives [1,2,3]. Issues with optimizing functionality are also linked with other commonly cited discomforts. Reducing prosthetic weight and retained socket heat typically requires designers to compromise on hardware used to deliver more robust performance [4,5]. Functionality, as defined by Light et al. [6], is the extent to which a device can adapt to perform certain tasks. Specifically for upper-limb prostheses, functionality can be classified at various levels: reliable grasp at the level of the hand, rotation at the level of the wrist and forearm, and movement in various heights at the level of the elbow and shoulder [6]. This research focuses on discovering methods to improve the most basic functionality, the grasp.

### 1.2. Background

Methods to address the issue of poor grasp prosthetic performance, without needing heavier or energy-intensive hardware components, are a priority. This research broadened the lens utilizing animal-based biomimicry. In general, biomimicry is defined as “innovation inspired by nature” [7,8,9]. This concept has been applied to several engineering applications. One field where biomimicry is commonly applied is the development of materials. Spider silk is known for its relatively high tensile strength and flexibility, which has prompted research on synthetically mimicking the unique material properties for mechanical testing [10,11,12]. The invention of Velcro^®^ was inspired by Xanthium strumarium (Cockleburr), which has spiky fruits called burrs that easily hook onto animal fur to spread [13]. The designer mimicked the structure of these burrs to design a material with hooks and loops that would securely latch onto itself while being easily removable [14,15]. Biomimicry also has applications in robotics [16,17]. To develop a robot suited for running on irregular terrain, researchers Clark et al. [16] drew inspiration from the morphology and material properties of a cockroach leg to design a robotic leg that would emulate a similar behavior. Biomimicry inspired by amphibians, such as sea snakes and salamanders, was applied to create robots that perform undulating motions to maneuver through the water [17]. The use of birds specifically as biomimetic inspiration for robotics has also been explored for a variety of applications. Robotics for flight is a popular topic of research. For mechanical wing systems, the behavior of birds in adapting to their wing morphologies has been mimicked to maximize aerodynamics under different flight conditions [18]. The leg and claw structures of birds are also a common inspiration for designing landing and takeoff mechanisms. Feliu-Talegon et al. [19] utilized bird-inspired structures to stabilize and facilitate the takeoff action of flapping-wing robots.

The concept of biomimetic design has been applied to prostheses, with most upper-limb prosthetic devices modeled after the human hand and arm. The research by Varol et al. [20] describes different biomimetic approaches to designing a human-inspired prosthetic. One approach follows the biomechanics of the human hand closely to model the overall structure of the hand and designate the placement of the actuators [20]. A second approach looks to modify certain characteristics to make aspects of the device more practical for robotic systems. These changes include the placement of the actuators in the hand rather than the forearm and setting the opening of the arm as the active motion [20]. Other examples of biomimicry aiding the development of upper-limb robotics include inspiration for sensors. Stretchable sensors for soft robotics have been inspired by the structure of mechanoreceptors in plant surfaces and the skin of humans and animals [21]. The structure of bird claws has also been used to design soft robotic grippers. The research by Wang and Yu simulated air pressure-powered grasping devices modeled after features in bird and human claws [22].

Applications of biomimicry also go beyond simple replication and rather utilize the underlying mechanisms of that process [8,20,23,24,25]. For prosthetic design, improving the ability of the device to reliably grasp different types of objects will be beneficial to the user. This investigation aims to apply biomimicry, not exclusively human hands, specialized for grasp-related purposes.

### 1.3. Applications

**Figure 1:**
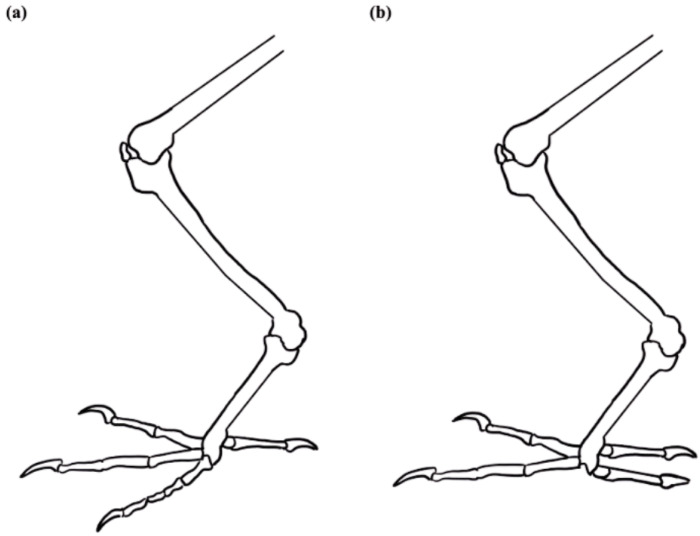
Representation of the two bird claw configurations found in nature: (**a**) anisodactyl; (**b**) zygodactyl.

The biomimetic model in this research investigated specifically how bird claws are suited for grasp-related applications and how they could improve the functionality of upper-limb prostheses. Across the many species, different birds have evolved unique adaptations to their claw structures that are suited for their environment and lifestyles. In identifying natural processes suited for prostheses, this investigation highlighted the claw structures of birds specialized for climbing or grasping. These structures are termed anisodactyl and zygodactyl claws and are displayed in Figure 1. Anisodactyl represents a configuration with three digits in the front and one at the back, and the zygodactyl represents a configuration with two in the front and two at the back [26].

The anisodactyl claw is commonly found in birds that perch and hunt their prey, for which the three digits in front offer greater shock absorption upon impact with structures or prey when approaching them at high velocities [27,28]. The outer digits on the front three act as stabilizers and provide additional reach for these actions [26]. Examples of birds exhibiting this pattern include birds of prey such as Falconiformes (falcons) and Accipitriformes (eagles and hawks) [26,28]. The zygodactyl claw is commonly found in birds that climb; the back two digits offer support and stability for a bird perched on vertical surfaces and allow improved maneuverability in this axis [26,29]. Piciformes (woodpeckers) and Psittaciformes (parrots) are among the many examples of birds exhibiting this claw configuration and behavior [29]. The objective was to design a representation of these digit patterns and to evaluate them for their grasping and mechanical abilities. To identify how to construct and evaluate these models, this research reviewed existing applications in the literature mimicking features from birds through computer-aided design (CAD) and 3D printing.

One such example is a study conducted at Stanford University, where researchers Roderick et al. [27,30] created a 3D-printed drone modification based on the leg and claw structures of birds of prey. The drones use these additions to land on irregular surfaces such as tree branches and carry various payloads. This allows them to rest and conserve battery during long-distance flights and to carry supplies for disaster responses. The mechanism of the drone attachment also goes beyond a similar morphological mimic of the bird’s legs and bases the features of actuation on a behavioral analysis of a bird performing a perching motion. Additional examples in the literature applying structures and mechanisms from bird claws in drone perching applications further corroborate the success of this aspect of biomimicry and aim to build on the grasping ability [26,31,32]. A study by Zhu et al. [26] tested some of the grasping and mechanical features of their developed claw model to assess its performance reliability. The design aims to transition the specific tree branch grasping application of the drone claw models to grasp a wider variety of objects. The model by Zhu et al. [26] could then be implemented for other applications and industries. This study tested two mechanisms of actuation of the claw: a standard model with the flexor digitorum longus and the extensor digitorum longus tendons and the other with a flexor digitorum longus tendon and torsion springs. Both types were evaluated based on their ability to adapt their grip to objects of varying sizes and incrementing weights.

A similar study by Nabi et al. [31] explores the utility of designing a robotic hand inspired by a pigeon claw, rather than a human hand, for industries that benefit from robotic automation. Specific features identified to streamline mechanical design include fewer actuators, reduction in material via fewer digits and joints, and a smaller palm size to improve the ability to maneuver smaller objects [31]. By expanding the initial scope of 3D-printed bird-inspired actuators, the literature supports how grasp-type devices modeled after bird claws may serve as an ideal inspiration for improving grasping devices.

To evaluate these grasping advantages, this research aimed to develop similar bird-inspired models for comparison with a traditional five-fingered prosthetic device. The Limbitless Solutions^®^ prosthetic hand was used as the model of comparison [33,34] and built upon the Roderick et al. [27,30] research framework. The biomimetic models of this design drew structural inspiration from the existing literature utilizing the biomechanical features from bird claws along with certain parts from the Limbitless Solutions^®^ five-fingered hand to translate the model from drone landing to prosthetic testing application. The design and assembly features of the biomimetic models and prosthetic hand were kept comparable to allow the testing to be more standardized. The biomimetic models were better standardized for evaluation alongside the five-fingered prosthetic. The Limbitless Solutions^®^ prosthetic hand was developed in the same lab as the bird-inspired models, allowing all devices to be constructed with similar manufacturing techniques. The developed models were not intended to serve as functional prostheses; instead, these claw models served as a framework to assess how biomimicry could offer insights into potential improvements to the current state of upper-limb prostheses. The tests conducted on the devices involved a mixture of the evaluations used for biomimetic models in the literature along with existing biomechanical load testing standards for prostheses. The aim was to better understand how the device would function under those conditions. Through both qualitative and quantitative analyses, the grasp performance of the anisodactyl and zygodactyl claws was compared to that of the transitional five-fingered prosthetic. The results of these comparisons inform specific features that could be implemented in future prosthetic design to improve grasp.

## 2. Materials and Methods

### 2.1. Design

**Figure 2:**
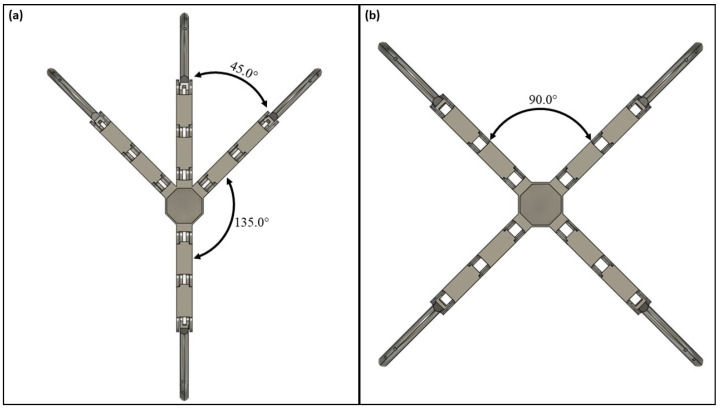
2D depiction of the assembled bird claws and digit configuration angle: (**a**) anisodactyl claw; (**b**) zygodactyl claw.

This work focused on developing a configurable multi-fingered mechanical model that mimics the anatomy and physiology of bird claws. Development primarily focused on two models with four digits, the anisodactyl and zygodactyl bird claw, characterized by key differences in digit arrangement. The anisodactyl and zygodactyl bird claw models are seen in Figure 2. Each digit on the frontal portion of the anisodactyl claw has an approximate angle of 45 degrees between the three digits, respectively. The outermost digits on the frontal portion of the palm and the rear digit have an approximate angle of 135 degrees.

Development of the zygodactyl base design adopted a similar approach. With a two-in-front and two-in-back configuration, each digit had a spacing of approximately 90 degrees. This created a symmetrical bird claw as seen in Figure 2b. This research designed a fully symmetrical bird claw design, a slight modification from a traditional zygodactyl bird claw seen in nature. Zygodactyl claws in nature (Figure 1b) do not follow a symmetrical digit arrangement compared to our zygodactyl model, which has equidistant digits. Biomimicry does not require the exact replication of biological elements found in nature and rather focuses on the functional aspects of the design [8,23,24]. This model was adapted to mimic the more symmetrical quality of the zygodactyl claw to evenly support a grasped object on all sides.

In nature, birds with these digit patterns often have differently sized digits on the same claw similar to how the fingers on a human hand differ proportionally [28]. These variations occur between different species exhibiting the same digit arrangement and even between members of the same species. The designed models kept the sizes consistent for digits by using the same phalanges and talons for each. The digit size variations may also depend on the dexterity of an actual bird claw for full effect, which a robotic application may not be able to truly replicate. Consistent digit sizes also facilitate the design and prototyping processes.

**Figure 3:**
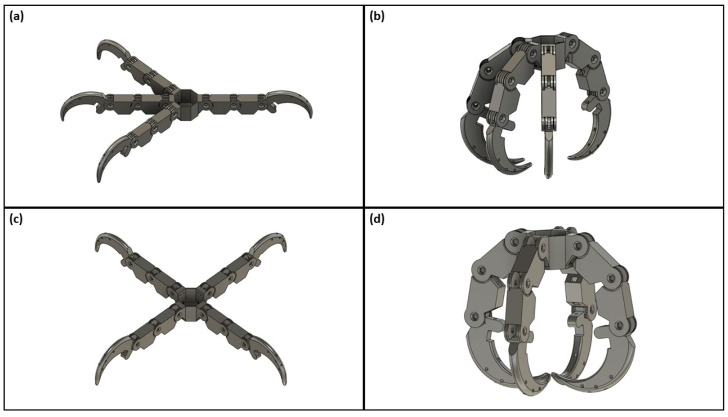
3D representation of an ideal grasp for both claws using the motion study analysis: (**a**) anisodactyl open claw; (**b**) anisodactyl closed claw; (**c**) zygodactyl open claw; (**d**) zygodactyl closed claw.

A preliminary digital model of the bird claw was designed in CAD software. This software allows users to conduct motion studies that depict the ideal movement of 3D structures moving in conjunction with one another. Ideally, the actuation of the claw will result in the proximal phalanges moving first, followed by the distal phalanges and talons relative to the claw base. The motion study feature was used to visualize the contraction of a bird claw starting from the open claw position to a fully contracted grasp as seen in Figure 3. Analysis of the motion study revealed crucial structural and alignment impediments with the bird claw design, which were modified as needed to optimize claw functionality. Following the completion of the digital CAD model, each component (base, phalanges, and talon) of the bird claw was 3D-printed with acrylonitrile butadiene styrene (ABS) plastic using fused deposition modeling (FDM) 3D printing. FDM 3D printing deposits melt plastic layer-by-layer in the geometry of the defined shape until a full 3D model is created [35,36]. In addition to printing the 3D model with the desired material (ABS plastic), Supplementary Materials is applied to reinforce susceptible geometries against warping during printing. These vulnerable geometries may encompass overhangs and cavities within the 3D model. This study used soluble support material, Stratasys SR-30, to facilitate the 3D printing process for each claw component. These models were 3D-printed with the use of a Fortus 250mc 3D printer and post-processed in an SCA-1200HT support cleaning apparatus. A support cleaning apparatus was used to dissolve any residual support material that remained on the 3D model after the manual removal of supports.

**Figure 4:**
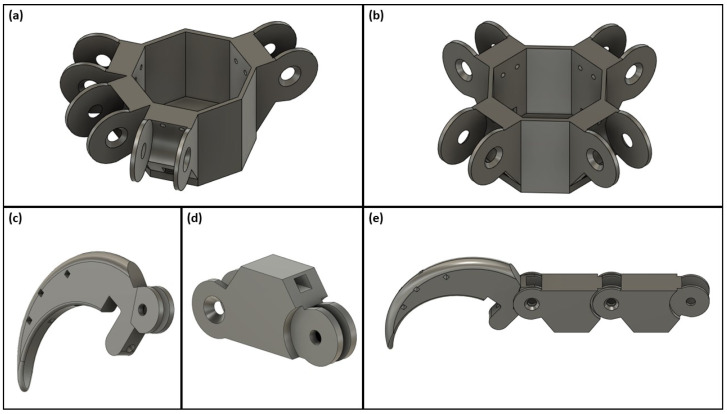
3D claw components used for assembly of a complete bird claw: (**a**) anisodactyl claw base; (**b**) zygodactyl claw base; (**c**) talon; (**d**) phalange; (**e**) fully assembled digit.

The bird claw was designed in three components: anisodactyl/zygodactyl base, phalange, and talon. Each component is shown in greater detail in Figure 4. A fully assembled claw consists of 1 base (either the anisodactyl or zygodactyl), 8 phalanges, and 4 talons, excluding the various joint and tendon parts that connect the components together.

**Figure 5:**
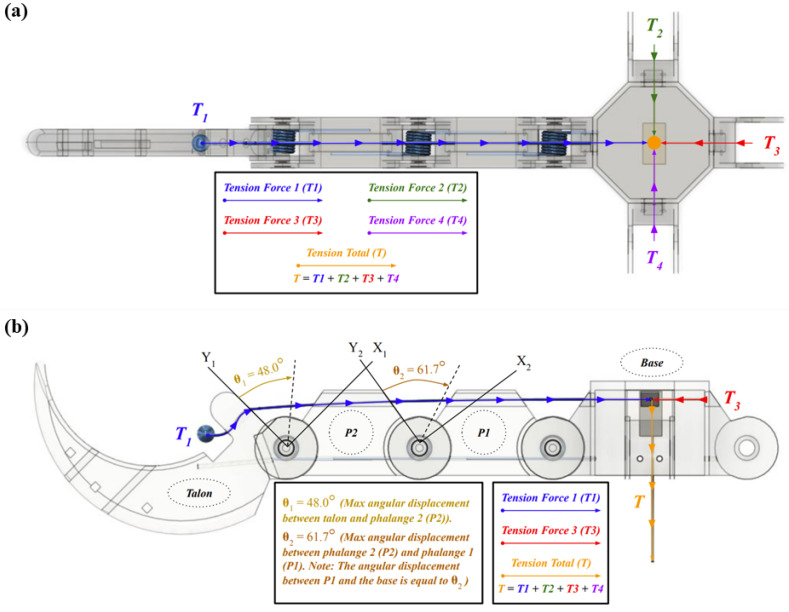
Single digit free body diagram: (**a**) top view; (**b**) side view.

Multiple design elements were derived from the Limbitless Solutions^®^ five-fingered prosthetic hand [33,34] and the Stanford drone model [27,30]. The foundational framework of the bird claw drew inspiration from the Stanford drone model, which primarily influenced the design of the phalange and talon [27]. This design provided a model of a tested bird claw that was developed to assess bird claw functionality in grasp and perching-related functions. These applications matched the goal of this investigation to assess the grasp and grip functions of bird claws, which can be referenced to improve current upper-limb prosthetic devices. Additional elements of the bird claw were modified in comparison to the drone model such as the joints. The Stanford team 3D-printed each joint of the phalange and talon as one unified digit. In contrast, this model created each phalange and talon separately, assembling the digit using finger posts and torsion springs as seen from the top view in Figure 5a. The choice to use finger posts was derived from the Limbitless Solutions^®^ five-fingered prosthetic hand to improve cohesion between a previously tested prosthetic hand and the bird claw model [33,34]. For mechanical testing purposes, developing a bird claw model that utilizes the same components found in a traditional prosthetic is crucial for direct comparison of functionality. Torsion springs were implemented into the joints of the digit to return the digit to its initial resting position after a full contraction is achieved. The resting configuration of the bird claw is characterized by an open palm structure, with all four digits in a relaxed position.

During the digit assembly process, both target phalanges are aligned to ensure their joints overlap. A torsion spring is secured between each joint to supply torsion force, and a finger post is inserted into the central overlapping hole to connect the two parts. The use of a finger post and torsion spring allows the digit to mimic the movement of a pin joint, which allows each phalange to rotate about a central axis while being restricted to one degree of freedom. A 0.61 mm stainless steel cable was used to mimic the tendons of the bird claw. Creating a four-bar linkage by connecting the actuator to the talon with the intermediate phalanges in between allows the digit to curl without interference [37]. Figure 5a displays the junction point that comprises the four-bar linkage where all four digit cables meet together. Once threaded through the digit, each cable was crimped at the distal portion of the talon as well as the main cable in the bird claw base. The cables threaded through each of the 4 digits were crimped together with the main cable, which was then coiled around a spool attached to a MG996R servo motor. The main cable serves as the central connection point for the four-digit cables at the base, ensuring consistent contraction of each digit. The main cable was fed through the base and secured to the spool at the top of the claw housing, controlled by the servo motor, which rotates to actuate the digits. To create a grasping motion, the servo motor winds in a clockwise motion, reeling the cable in to curl the digit inward. To release the grasp, the servo rotates counterclockwise to relax the wound cables, and the torsion springs return the claw to its starting position. Figure 5b highlights the maximum angular displacement of each digit component during actuation. Maximum angular displacement represents how far each digit component can rotate before parts of their body begin to interfere with one another. The talon and the second phalange are capable of rotating 48.0 degrees relative to one another before a component of the talon begins to contact the face of the second phalange. These exact values were determined with the same motion analysis study used to analyze the ideal actuation of the fully assembled claw. These values reflect the ideal actuation of a singular digit. The angular displacements are significantly reduced during a grasp due to interference caused by other digits.

**Figure 6:**
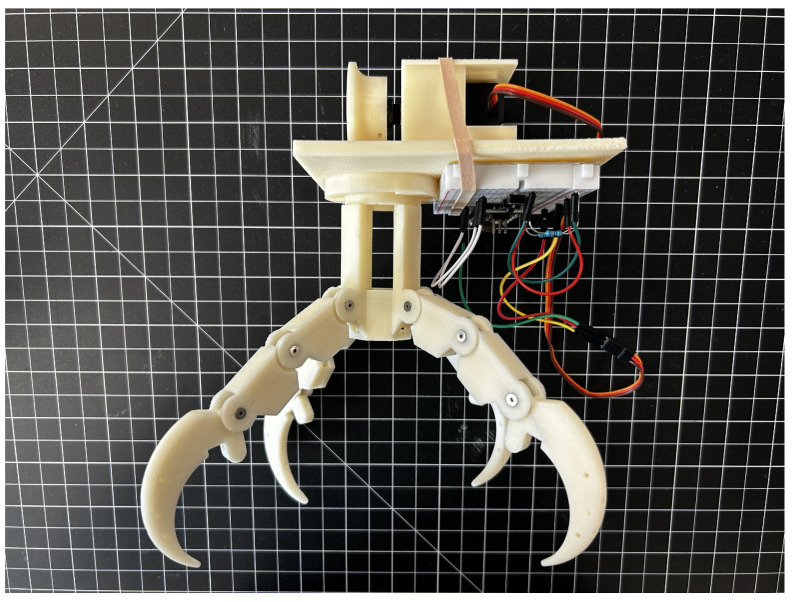
Fully assembled zygodactyl claw positioned within the prosthesis model housing, including the MG996R servo motor and two-button breadboard circuit.

In addition to the components required to actuate the bird claw, various devices are required to control the servo motor and house the various bird claw parts. The housing encloses the servo motor, cable spool, a two-button breadboard circuit, and a detachable swivel base as seen in Figure 6. The two-button breadboard circuit consists of an Arduino Nano programmed using the Arduino Integrated Development Environment to control the rotation of the servo motor through two buttons. Pressing the first button would rotate the servo motor 180 degrees clockwise to wind the cable in to curl the claw digits, and pressing the other button would reset the motion by rotating the servo 180 degrees in the other direction. The housing design also includes a custom swivel base which allows the different claw configurations to be easily inserted and removed for mechanical testing and repairs.

### 2.2. Testing Methods

#### 2.2.1. Qualitative Assessment

**Figure 7:**
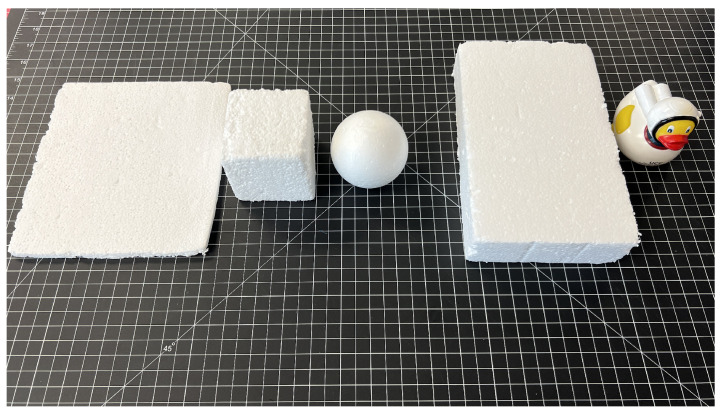
Objects used in qualitative assessment, from left to right: thin (220 mm × 135.5 mm × 48.5 mm), cubic (75.0 mm × 75.0 mm × 75.0 mm), spherical (75.0 mm diameter), wide rectangular (225.0 mm × 135.5 mm × 48.5 mm), and irregular (L_max_ 74.5 mm × W_max_ 180.0 mm × H_max_ 66.5 mm).

With the constructed anisodactyl and zygodactyl models, physical evaluations were executed to identify the biomechanical advantages of designing the grasping devices after two different four-fingered bird claws versus the traditional five-fingered human hand. The first phase involved a qualitative experiment assessing the grasp interactions of the three different devices: the anisodactyl claw, zygodactyl claw, and Limbitless Solutions^®^ prosthetic. This experiment utilized lightweight objects of various shapes and sizes to assess how each device established a grip for grasping varying topologies. This experimental procedure was modeled after the methods of Zhu et al. [26], who tested the efficacy of two different anisodactyl claws adapted to objects establishing a secure grip. The objects chosen for this experiment are displayed in Figure 7: plane, cubic, spherical, wide rectangular, and irregular shapes. The first four shapes were created using Styrofoam^TM^ to minimize potential grasping differences due to object weight. The final irregular object, represented by a rubber duck, was introduced to assess the device’s approach to a more complex grasping of an uneven geometry. The procedure for this evaluation required the device to pick up the object from a resting position, hold it for at least five seconds to ensure a successful grasp, and then release the object. Evaluating device interactions with each of these objects offered insights into whether the biomimicked designs held any advantages in grasping specific shapes and sizes.

#### 2.2.2. Quantitative Assessment

In addition to assessing object grasping, performance loading was evaluated. Specifically, the biomimicry designs were evaluated under prosthetic testing conditions for single-digit flexion and full grasp tests [38,39]. The results of tests under these conditions indicated the biomechanical advantages or disadvantages of structuring a prosthetic device with the bio-inspired designs. Modifications under consideration were the reduction in number of digits, the display of digits relative to the palm, and the shape and sizes of the phalanges and talon components [38,40]. The components under evaluation included the 3D-printed parts of the base and digits and the assembly parts including the finger posts, torsion springs, cable, and crimps. The circuit and motor driving the actuation were omitted from the load testing since the focus of this assessment was to test the ability of the plastic and connecting components to withstand the specified loads. The electromechanical actuation system’s primary purpose was to drive the grasp and release of the claw for the quantitative assessment, and the MG966R servo motor used is not necessarily reflective of the actuator found in a functional prosthetic device.

**Figure 8:**
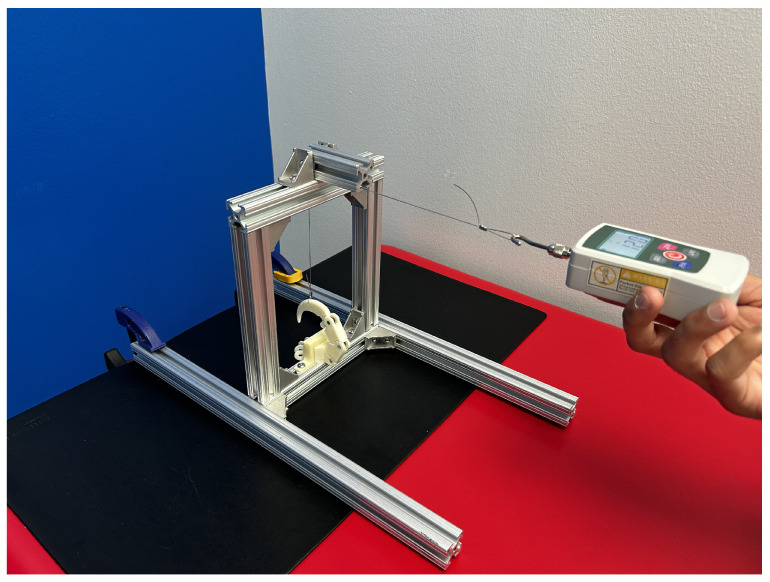
Single-digit flexion testing apparatus displaying the secured digit and the force application measured through the handheld force gauge.

The single-digit flexion test, informed by the testing procedure carried out by Mio et al. [38], evaluated the load capacity for a single digit [38,39]. The digit extension test conducted by Mio et al. follows guidelines outlined by the plastic testing standards ISO 178 and ASTM 790 [41,42]. The existing standards for prosthetic devices typically have the finger fully extended to apply a load at the most distal part of the digit, serving as a correlate for the pinch force capacity of a single finger [38,39]. The minimum load to meet this test’s requirement is 30N [38,39]. The test conducted on the biomimicry models was modified to start with the digit in a curled position. The deviation was minor, due to the geometric differences between the talon and prosthetic finger design. This modification was made since the developed bird-inspired models do not use pinching as the primary grasp method, whereas the original testing procedure was designed to measure the prosthesis’s pinch ability [38,40]. The force applied to the digit in a curled starting position was more similar to the type of force the digit would encounter when grasping an object. The single digit was mounted to the testing platform and secured as shown in Figure 8. The cabling running through the channels in the digit was pulled taut and crimped at the level of the base and talon to retain the digit in its curled position. An upward force was applied to the talon to represent the force encountered by a grasped object due to gravity. The loaded cable was looped over the t-slot bar at the top of the testing platform and pulled vertically. A handheld digital force gauge was used to simultaneously pull the cable and measure the force in newtons until the digit failed. Failure in this case was defined as the inability of the digit to retain its original position. The failure condition, defined as extreme digit displacement, caused the cable, finger posts, or 3D-printed plastic to break or deform.

**Figure 9:**
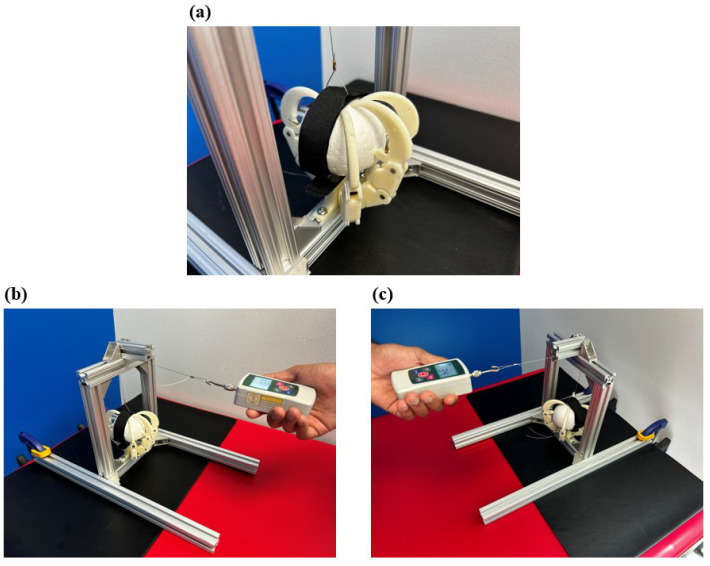
Full-claw flexion testing apparatus: (**a**) close-up view of the test object placed in the grasp of the anisodactyl claw; (**b**) load test conducted on the anisodactyl claw; (**c**) load test conducted on the zygodactyl claw.

The full grasp test, following the prosthetic testing standards outlined in Mio et al. [38], assesses the ability of the full hand to support a load. This portion of the work by Mio et al. follows the test procedures outlined in ISO 22523 [43]. The minimum requirement for this standard is 60 N [38,39]. The device was supplied with an object, and a force was applied to the object to represent the weight of a grasped object. The applied force was measured until the device’s failure, similar to the failure criteria of the single-digit flexion test. For the grasping object, a sphere was chosen due to its grasping reliability by both the zygodactyl and anisodactyl claw patterns. The sphere was adapted by removing its lower half and flattening the bottom so it would rest better on the base and mounting clamp. The object was placed in the grasp of the four-fingered device and enveloped by a Velcro^®^ band to serve as an attachment point for the cable used for force application, depicted in Figure 9a. For both devices, a consistent increase in load was applied to the object until the failure of the device to retain the object within its grasp. The testing platform for the anisodactyl claw is displayed in Figure 9b and the zygodactyl claw in Figure 9c.

## 3. Results and Discussion

### 3.1. Qualitative Grasp Test Results

**Figure 10:**
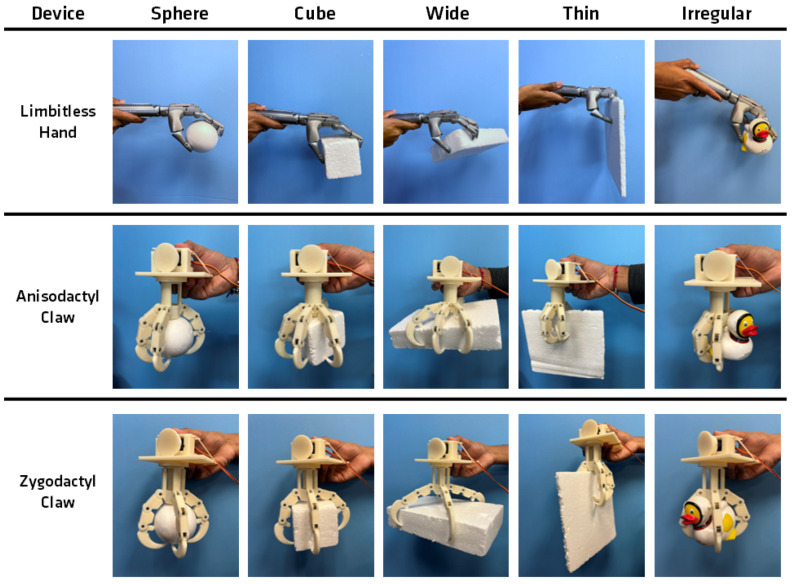
Qualitative grasp test results displaying the grasp interaction between each device and object type.

In observing how each device interacted with the various objects as noted in Figure 10, key features from each of the devices that allowed for an improved grasp were identified. All three devices executed the intended function of contracting the more proximal joints followed by the distal joints relative to the base when grasping [44]. This was evident by the pattern in which the devices initiated contact with the objects. The phalanges closest to the palm made contact with the object, allowing the other sections to wrap around the object to establish more points of contact along the object. This enabled the digits to distribute the force rather than rely on a pinch-type grasp where a greater amount of force is concentrated at fewer points of contact.

A structure on both the biomimicry claws that emphasized this grasping method was the smaller, more central palm size. This feature improved opportunities for the more proximal portions of the digits to contact the object. Existing bird-inspired grasping devices with small palm structures similarly experience increased contact points between the digits and grasped objects [26]. The five-fingered hand, which has a much larger palm not located as central to the device, relied on grasping with more distal portions of the fingers in certain cases when not all of the proximal parts of the fingers could reach the object. As seen in the sphere column of Figure 10, the more central palm in both the anisodactyl and zygodactyl bird claw created a more secure grip around the object as opposed to the more human hand. These differences in grip were due to fewer points of contact seen in the Limbitless Solutions^®^ hand. The primary grasp force is developed between the index and middle fingers and thumb, leaving a large portion of the palm exposed during a grasping motion. These factors led the grasp to be more pliable and the object easier to dislodge in the five-fingered hand. Although the palm of an actual human hand is similarly large, its composition allows it to deform and wrap securely around a grasped object. A similar effect may not be feasible to achieve for a low-cost prosthetic comprising rigid materials. Therefore, reducing the palm size to allow the digits more access to larger objects appears to be one solution for improving the grasp while maintaining a simpler design.

Another feature of the biomimicry claws that supplemented a more secure grasp was the structure of the most distal digital component: the talon. As the talon was curved, it offered additional reach in the curled position of the digit to make contact with the sides of wider objects. The five-fingered hand encountered difficulty in grasping all the way across the wide object and instead relied on making contact with adjacent sides, leading to a less stable grasp. Prosthesis with multi-positional thumbs may be able to change grip configurations to mitigate these limitations. The curved talon also served as an enclosure for smaller objects such as the sphere and irregular shapes by converging at the base of the object. The talon encountered challenges when grasping the thin object where the curved structure often forced the sharper digit tip to embed into the material to be able to establish a grasp. Predatory birds often use their talons to puncture prey. Grasping in this fashion may be problematic when handling fragile objects. In cases when the talon tips did not embed into the object, the contact from the relatively thin sections of the talon was precarious. The narrow contact afforded by talon-shaped digits is also observed in the study by Zhu et al., where the bird-inspired models faced similar challenges with thin geometries [26]. In comparison, the flatter, more linear fingertip of the five-fingered hand performed well in establishing a better grip on the thin object with a better pinch grip pattern. The pinch grip of the five-fingered hand offers a more reliable grasp for thin objects, whereas the encompassing grip of the bird claw models is generally preferred for larger objects [44]. A pinch grasp still demonstrates success in grasping larger objects; however, the encapsulating grasp of the bird claw models may be better suited for grasping larger objects in comparison to the five-fingered hand.

When comparing the two claw types, certain advantages exist between the anisodactyl and zygodactyl structures. The anisodactyl claw with its separated front and back digits is well suited to grasp objects that typically only require support on two sides. The rectangular shape benefits from this grasp due to its different length and width proportions. This also applies to cylindrical shapes, which corroborates why much of the literature focusing on bird claws for drone applications mimics an anisodactyl structure perching on cylindrical tree branches [27,30]. The zygodactyl claw, which was adapted to be radially symmetrical, establishes a more secure grasp with objects with more uniform sides such as the sphere and the block. The symmetrical display creates points of contact more evenly across the faces of the object. The larger spaces between the front and back digits in the anisodactyl claw create more of an opportunity for these types of objects to be dislodged from the grasp. A similar grasp feature was observed in the five-fingered hand due to the digits being positioned in the front and side of the palm, making it easier to grasp cylindrical objects with more direct contact from the digits as opposed to the zygodactyl model.

### 3.2. Quantitative Grasp Test Results

For the single-finger digit flexion test, the force was applied by pulling on the cable attached to the talon segment of the claw in its curled position. Recording the applied force with the handheld digital force gauge, the single digit consistently supported loads past the standard of 30 N for a mechanical prosthetic finger [38,39]. Across the five load assessments conducted for the single digit, the average load was 61.0 N, with a tolerance of 2.2 N for one standard deviation from the average. The maximum load the digit could support before failure was measured at 64.3 N, with failure occurring due to the cable in the curled digit snapping. Repeated testing indicated that the break generally occurred at the level of the crimp at the talon preventing the cable from passing through this channel. Supporting the cable at the talon crimp may help withstand further loads for a single digit. This test indicated that a 3D-printed digit structured after the morphology of a bird claw matched and even exceeded mechanical load requirements for prosthetic digits. The average load and tolerance for the five trials also demonstrated that the results were fairly consistent between trials. When observing the digit as it was being pulled, the curvature of the talon allowed the phalanges to straighten while retaining the position established where the pull cable was attached. Verification of the biomechanical load-bearing capacity of this specific structure is important in discovering whether it has any apparent weaknesses that would disqualify it for adaptation into prosthetic design or whether it holds any novel advantages, despite this current prototype not acting as a functional prosthetic.

For the full grasp test, separate tests were conducted for both the anisodactyl and zygodactyl configurations, and results were compared to existing prosthetic standards to determine whether the structure of either had advantages in supporting more load. The expectation for a full grasp test is to support 60 N of pulling force [38,39].

The first test with the anisodactyl model demonstrated this configuration supported a maximum of 86.1 N as an assembly. This result also exceeds the maximum load supported by any single digit (64.3 N), which is expected as there are additional digits to distribute the applied force [45]. However, the average for the five trials was lower at 76.8 N, with a tolerance of 9.0 N. Compared to the values for the single digit test, the results were not as consistent for the full anisodactyl grasp. The failure condition for this test occurred when the outer sets of digits on the front three digits partially relaxed from the deformation of the cable retaining the curled position of the claw. The digit relaxation allowed the object to escape the grasp through the gap between the front and back sets of digits. An explanation for the larger deviation in results may be in the difference in applying loads with an object rather than directly to the fingertip. The specified failure condition of the object slipping likely introduced some additional variability that may not occur when the load is applied directly via a cable and not the objects for the single digit.

The zygodactyl grasp test took several attempts to reach a maximum load that surpassed the 60 N threshold for a full grasp test. In the first two pull tests, it was observed that the cable adjacent to the crimp at the level of the talon tended to snap or deform, much like the failure condition for the single-digit extension test and the anisodactyl full grasp test. One possible explanation for the difference in performance between the two configurations is the lack of digits aligned with their respective cables in the zygodactyl testing setup. This may have caused the force to be applied at an angle to the digits and the cables holding them in place, resulting in the force overcoming the crimp at the talon with more ease. Tightening the cables in the claw to better position the crimps to avoid them adopting an unnatural angle helped overcome this issue, with this trial meeting a maximum pull force of 74.1 N to exceed the 60 N threshold. Repeat tests with this consideration for the zygodactyl claw demonstrated more consistent results in meeting the 60 N threshold. The average for the five trials was 58.0 N, with a tolerance of 10.2 N. Though the maximum values exceeded the 60 N requirement, the average is lower due to the premature failures observed in the first two tests. The results of each test are displayed in Table 1.

In comparing the results of the three different tests, it is observed that the maximum applied loads for the single digit significantly exceeded the minimum requirement. However, the maximum loads for the full claws were only higher by approximately 10–20 N. The device’s assembly components, rather than the 3D-printed plastics, may have been the driving factor in the load the design could support. Adding more digits did not proportionally increase the supported load in comparison to the single digit since the failure condition included the cable or crimp deforming or snapping. Therefore, even one digit failing for the anisodactyl and zygodactyl patterns would likely lead to the object escaping the grasp. For the zygodactyl claw, the symmetrical arrangement of the digits may have been a disadvantage in this aspect since the loss of a digit on one end leads to an imbalance in the device’s support of the grasped object. This imbalance is also applicable for the single digit in the rear of the anisodactyl claw. In contrast, the anisodactyl claw may still retain an object if the digits in the front supplement the failure of a neighboring digit. This may explain why the average and maximum measured loads for the anisodactyl evaluation were higher than the zygodactyl.

### 3.3. Limitations

A key limitation in the qualitative grasp testing portion of the comparison between the biomimicry and five-fingered models was that the five-fingered hand had a silicone segment at the fingertip, whereas the talons of the biomimicry models did not. Talons are often used by birds of prey when hunting for kinetic puncturing of objects. The purpose of the use of silicone on the prosthesis is to create a larger surface area and higher friction force between the object and the fingertip. Silicone was not included in the design of the talon tips of the biomimicry claws to more closely mimic the biological structure of a talon, although this difference between the three devices may contribute to the grasp performances.

While the reference quantitative testing method from Mio et al. [38] used a Zwick Roell Z0.5 multi-test machine to apply a consistently increasing load to their models, this study relied on a human to apply an increase in load. This may cause the results to be more sensitive to inconsistencies in the rate applied. The average loads for the full grasp test reflect this limitation, especially for the zygodactyl full grasp evaluation. However, the testing procedure primarily evaluated whether the models were able to handle the minimum load requirements rather than understanding the exact load that leads to failure. Further development of this research may focus on the use of industrial load testing equipment to corroborate the preliminary grip force results with a more sensitive testing procedure.

## 4. Conclusions

To develop novel biomimetic solutions to address the issue of high prosthetic rejection, a biomimetic grasping assembly was developed with inspiration from bird claws to test for features that would be advantageous in improving grasping ability. The biomimetic sources, the anisodactyl and zygodactyl configurations found in bird claws, were chosen for their alignment in nature with grasp-related activities. Design inspiration was derived from existing applications in the literature, adapting a 3D-printed bird claw for drone landing attachments and upper-limb human-hand-based prosthetic design. A testing platform was created for both claw configurations to emulate the structural and behavioral characteristics of a bird claw in a 3D-printed device. The constructed devices were evaluated both qualitatively for how they interact with various objects and quantitatively for how much load they can support relative to the standards for upper-limb prosthetic devices. These evaluations indicated certain features that improve the grasping ability without compromising the mechanical load-bearing capabilities of the device.

1. The cable attachment points and torsion springs placement along the digits allowed for rotation of the most proximal sections of the digits inward followed by the more distal components. This pattern appeared more natural and was advantageous in securely grasping larger objects by wrapping the digits around them rather than pinching them by initiating contact with the fingertips.
2. The palm design of the bird-inspired device, with a smaller and more central palm relative to the digits, compared to the human-hand prosthetic improved the ability of both the biomimetic claws to grasp and hold the various objects. This design feature enabled the proximal portions of the digits to have a better opportunity to establish contact with the object to improve the overall grasp.
3. The curved talon at the distal end of the digit held benefits in offering support for wider objects. For objects small enough for the digits to wrap around them, the curved talons also acted as an enclosure for the object by converging at the base to reduce the chances of accidentally dropping it. A disadvantage of this feature was the difficulty in securely holding thin objects because of the thin contact point and reduced application of force due to the curved tip angle.
4. Although a pinch-type actuation for the five-fingered hand was observed to grasp larger geometries with less security, it demonstrated greater success in holding thin objects as observed in the object interactions with the five-fingered device. The flatter fingertip shape of the human-inspired five-finger hand further contributes to handling thin objects relative to the talon structure of the bird models by increasing surface area at the points of contact.
5. In comparing the anisodactyl and zygodactyl configurations, each digit setup had grasp advantages for certain object types. The anisodactyl’s 3 × 1 structure allows a wider grasp for more cylindrical objects. The central front and back digits create the primary grasp, the outer two digits in the front supply greater reach, and the greater angle between the front and back digits allows for the object to not be obstructed by another digit. The more symmetrical 2 × 2 feature of the zygodactyl allows an advantage when grasping smaller, more symmetrical objects as it more evenly supports the object on multiple sides and reduces the space between the digits for the object to potentially slip out.
6. Mechanical load testing results indicate that the robustness of the assembly is validated by testing standards traditionally applied for upper-limb prostheses and plastic properties. The maximum recorded load values were 64.3 N, 86.1 N, and 74.1 N for the single digit, anisodactyl claw, and the zygodactyl claw models, respectively. These values exceed the load metrics of 30 N for a single digit and 60 N for a full grasp set for prosthetic testing. The low averages for the full grasp tests across five trials warrant further testing with a mechanized force application procedure to further validate the initial results.

### Future Research in Upper-Limb Prostheses

These features have the potential to be implemented in the design of current prosthetic devices to make them more functional and reliable. Models may benefit from a design that strikes a balance between the features of the current human hand prosthetic and the developed biomimetic features. On current prosthetic hands, examples of this design approach may include resizing the palm to have it take up less space and positioning it more central to the displayed digits. It is also important to consider the existing advantages of the five-fingered hand, notably the pinch grip pattern that demonstrates improved grasping ability for thin objects, and ensure that any design modifications preserve those as well. Human-inspired hands are anticipated to be less task-specified and required to be adaptable to a variety of situations.

Adding a slight curve to the curvature of the fingertips may allow the fingers to establish a pinch-type grasp for thinner objects while still offering some additional reach. This testing enabled an adaptable research platform to be designed, enabling a variety of digit configurations to be feasible for testing. Modifications may be made to the current base to allow the digit to rotate around the central palm structure and enable different angular spacing between each digit.

In the effort to develop an optimized model to mitigate prosthetic rejection, future work would also benefit from exploring techniques to create more lightweight devices. New models with the mentioned modification may be constructed with different materials and evaluated to determine the properties that allow for a durable yet lightweight device. These mechanical evaluations may be assessed with finite element analysis (FEA), a simulation study run on physical models to assess how a model behaves under certain stresses. Next-generation models closer to a functional upper-limb prosthetic would also benefit from involving a wider variety of shapes in the qualitative evaluation; while the current testing evaluated the grasping ability of relatively elementary shapes for the initial models, updated designs would involve objects encountered in daily activities with more complex geometries to better inform how the device may perform for a prosthetic user.

This research primarily focused on the assessment of the anisodactyl and zygodactyl bird claw configurations; however, many different bird claw configurations exist in nature, including the heterodactyl, syndactyl, and pamprodactyl, each with their unique digit configurations and adaptations [26]. Future research investigating the utility of biomimicry from further types of bird claws in prosthetic design would be of use to uncover the most optimal digit arrangements and spacing angles for prosthetic use.

Further design modifications may also address a limitation between the existing prosthetic hand model and the 3D-printed test devices. The inclusion of silicone along the contact points may provide increased performance for all varieties. As observed in the qualitative grasp tests, the increased friction between the silicone and the object’s surface allowed the prosthetic hand to successfully grasp and hold objects even with fewer contact points. Including silicone along the major contact points of the developed bird claw models, notably at the interior side of the phalanges and the talon tip, may improve overall grip. Further increases in the prosthesis contact patterns may translate to improved functionality with limited weight increases. Further evaluating the performance, weight increase, and complexity for assembly may offer insights into optimization for the prosthetic version and the bird-claw biomimetic device.

## Figures and Tables

**Table 1:** Summary of the quantitative load testing results.

Test Type	Source [38,41,42,43]	Minimum Expected Load	Average Measured Load	Maximum Measured Load
Single-Digit Extension	Mio et al. (2019) ISO 178, ASTM 790	30 N	61.0 N ± 2.2 N	64.3 N
Anisodactyl Claw Grip	Mio et al. (2019) ISO 22523	60 N	76.8 N ± 9.0 N	86.1 N
Zygodactyl Claw Grip	Mio et al. (2019) ISO 22523	60 N	58.0 N ± 10.2 N	74.1 N

## Data Availability

Data is contained within the article or Supplementary Materials.

## Supplementary Materials

The following supporting information can be downloaded at: https://www.mdpi.com/article/10.3390/biomimetics9040195/s1, which includes the mechanical testing statistical analysis.

## Abbreviations

The following abbreviations are used in this manuscript:

AM	Additive manufacturing
FDM	Fused deposition modeling
ABS	Acrylonitrile butadiene styrene
CAD	Computer-aided design
FEA	Finite element analysis
